# Data on effect of NPSB fertilizer rates on growth and yield of carrot (*Daucus carrota* L.) varieties in Gondar district, Northwestern Ethiopia

**DOI:** 10.1016/j.dib.2024.111232

**Published:** 2024-12-16

**Authors:** Abebaw Mulugeta Andualem, Yenus Ousman Kemal, Derajew Asres Mekonen, Fentahun Asrat Yenet, Hulushum Woreta Kassa

**Affiliations:** a Department of Horticulture, College of Agriculture and Environmental Sciences, University of Gondar, Po Box.196, Ethiopia; b Department of Horticulture, College of Agriculture, Food and Climate Sciences, Injibara University, Po Box: 40, Ethiopia; c Department of Horticulture, College of Agriculture and Environmental Sciences, Debre Markos University. Po Box: 269, Ethiopia

**Keywords:** Dataset, Growth, Carrot, NPSB, Varieties, Yield

## Abstract

Carrot (*Daucus carota* L.) is one of the most important root crops grown worldwide and in Ethiopia. However, its production and productivity are low due to a lack of improved varieties and unbalanced fertilizer rates, among other factors. The field experiment was, therefore, conducted to improve the productivity of carrot varieties through blended fertilizer rates at the horticulture demonstration site of the College of Agriculture and Environmental Sciences, University of Gondar. Treatment consisted of NPSB nutrient levels (0:0:0:0, 21.3:15.3:2.80:0.04, 42.6:30.6:5.60:0.08, 64:46:8.41:0.12, 85.3:61.2:11.2:0.16, and 106.7:76.7:14.03:0.20 kg ha^−1^), which were laid out in a randomized complete block design with three replications. Data were collected on crop phenology, growth and root yield (marketable, unmarketable, and total root yield) parameters and subjected to analysis of variance using SAS computer software version 9.4. As described in Montgomery, the residuals were examined to verify the normal distribution and homogeneous variance model assumptions on the error terms for each response variable. Because the twelve treatment combinations were randomized within each block, the independence assumption is valid. When a treatment effect was significant, multiple means comparison was performed at a 5% level of significance using the least significant difference (Fisher's LSD) method to generate letter groupings, and correlation analysis was performed using the Pearson correlation procedure found in SAS. This dataset article, therefore, gives information about the effects of NPSB on the productivity of carrot varieties. The experiment also determines the optimal NPSB fertilizer rate for maximizing carrot root yield in comparable agro-ecological regions, offering valuable insights for researchers to analyze and potentially sparking new research directions. This data can foster collaborations and enhance the visibility of research within the scientific community, enabling widespread access for further exploration and application in related fields.

Specifications Table

**Table:** 

Subject	Agriculture
Specific subject area	Horticulture
Type of data	Raw, Analyzed, Table and Figures
Data collection	*Days to maturity, plant height, leaf number, root fresh weight, root length, root diameter, root volume, root dry matter, above ground biomass, marketable, unmarketable and total root yield* were obtained as described in this article.
Data source location	University of Gondar, Central Gondar, Ethiopia, is the owner of the data presented in this article. The experimental sites is located at longitude of 12°28’N, the longitude of 37°29’E and has an altitude of 1977 m.a.s l.
Data accessibility	Repository name: Mendeley data doi:10.17632/pny2rbrkpx.2 Direct URL to data: https://data.mendeley.com/datasets/pny2rbrkpx/2
Related research article	‘none’

## 1 Value of the Data

• These data offer a comprehensive evaluation of how different NPSB fertilizer rates impact carrot varieties in Ethiopia's Gondar district. This information is crucial for agricultural researchers looking to improve nutrient management strategies and enhance crop productivity.
• The dataset provides a robust foundation for comparative studies and meta-analyses, enabling scientists to examine fertilizer responses across various environments. This could facilitate broader insights into optimizing NPSB use for carrot and other vegetable crops in similar agro-climatic zones.
• Researchers in precision agriculture can leverage these data to refine predictive models for crop performance based on NPSB inputs, helping drive more accurate fertilizer recommendations that increase yield while reducing environmental impact.
• This dataset can be used as a benchmark for future studies conducted in different regions and seasons, enabling the creation of location-specific fertilizer management strategies to ensure optimal crop performance.

## 2 Background

Carrot (*Daucus carota* L.) is a biennial plant in the Apiaceae family, formerly known as Umbelliferae, valued for its high carotene content in the root. It grows as an annual crop for root production and is moderately hardy, thriving in cool conditions. Carrots are a vital vegetable, rich in alpha and beta-carotene, essential for vitamin A production. They are known for their nutritional value, including fiber, vitamin K1, potassium, and antioxidants, promoting weight loss and improving eye health. Carrots are popular globally and economically important due to their low cost, ease of production, and long storage life [1].

Carrots are a significant contributor to the world vegetable trade, with production areas expanding worldwide. The world annual production of carrots and turnips is substantial, with China leading in production. In Ethiopia, carrots are cultivated both for family consumption and income generation, with specific regions showing significant production levels [2].

The growth and yield of carrots are influenced by fertilizer application and variety selection, highlighting the importance of proper nutrition management for successful carrot cultivation. The application of NPK fertilizer at recommended rates ensures successful carrot yield. Harammaya-I and Nantes varieties benefit from 100 kg DAP and 100 kg Urea split application for root production [3]. N, P, S, and micronutrients play crucial roles in enhancing photosynthesis, growth, and metabolic processes for optimal carrot development [4]. Therefore, growers are in dire need to increase production, productivity, and quality to meet the potential market demands. As a result, it is mandatory to improve its productivity with the supplementation of different agronomic practices and identifying adaptable varieties for a study area.

## 3 Data Description

**Fig. 1:**
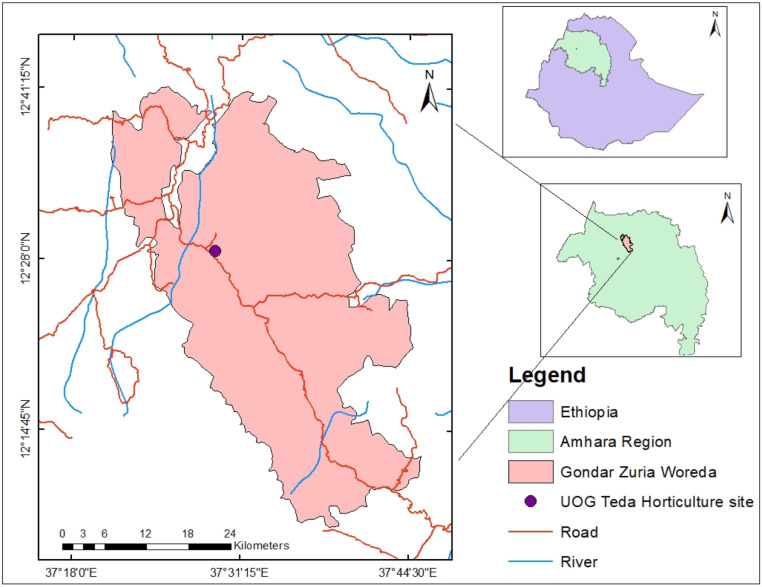
Geographical location of the study area, Teda, Gondar zuria, Ethiopia.

**Fig. 2:**
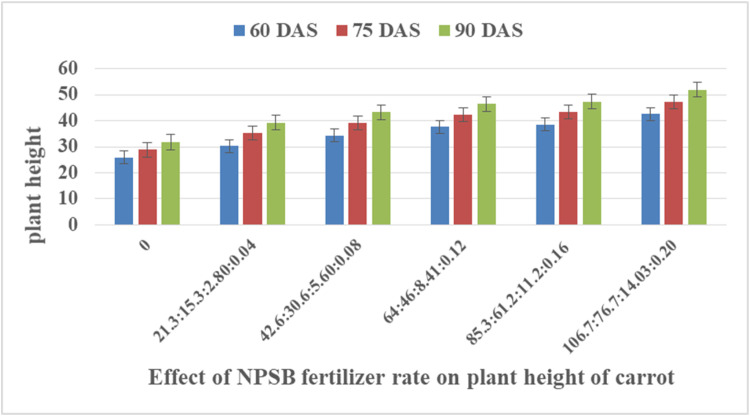
Effects of blended NPSB fertilizer rates on plant height between days after sowing (DAS).

**Fig. 3:**
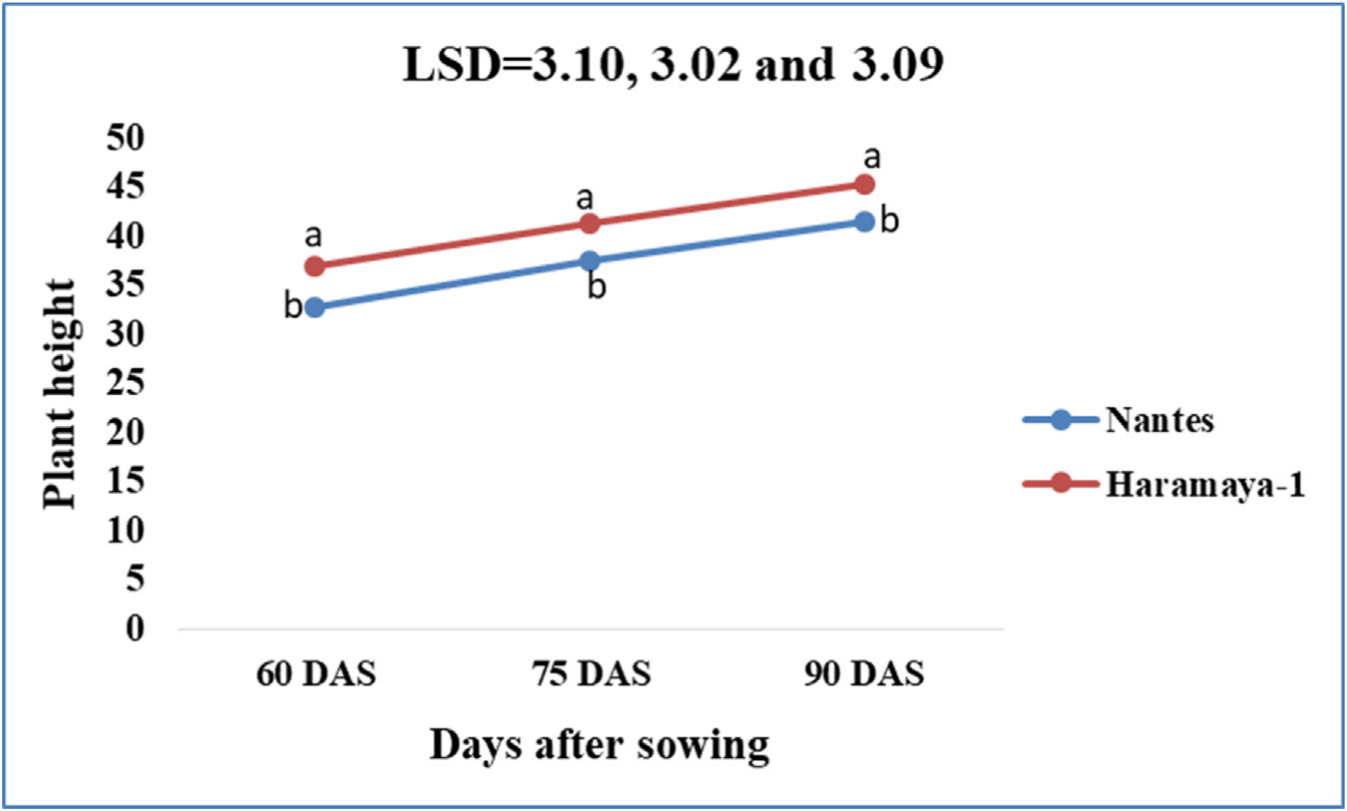
Effects of carrot varieties on plant height.

**Fig. 4:**
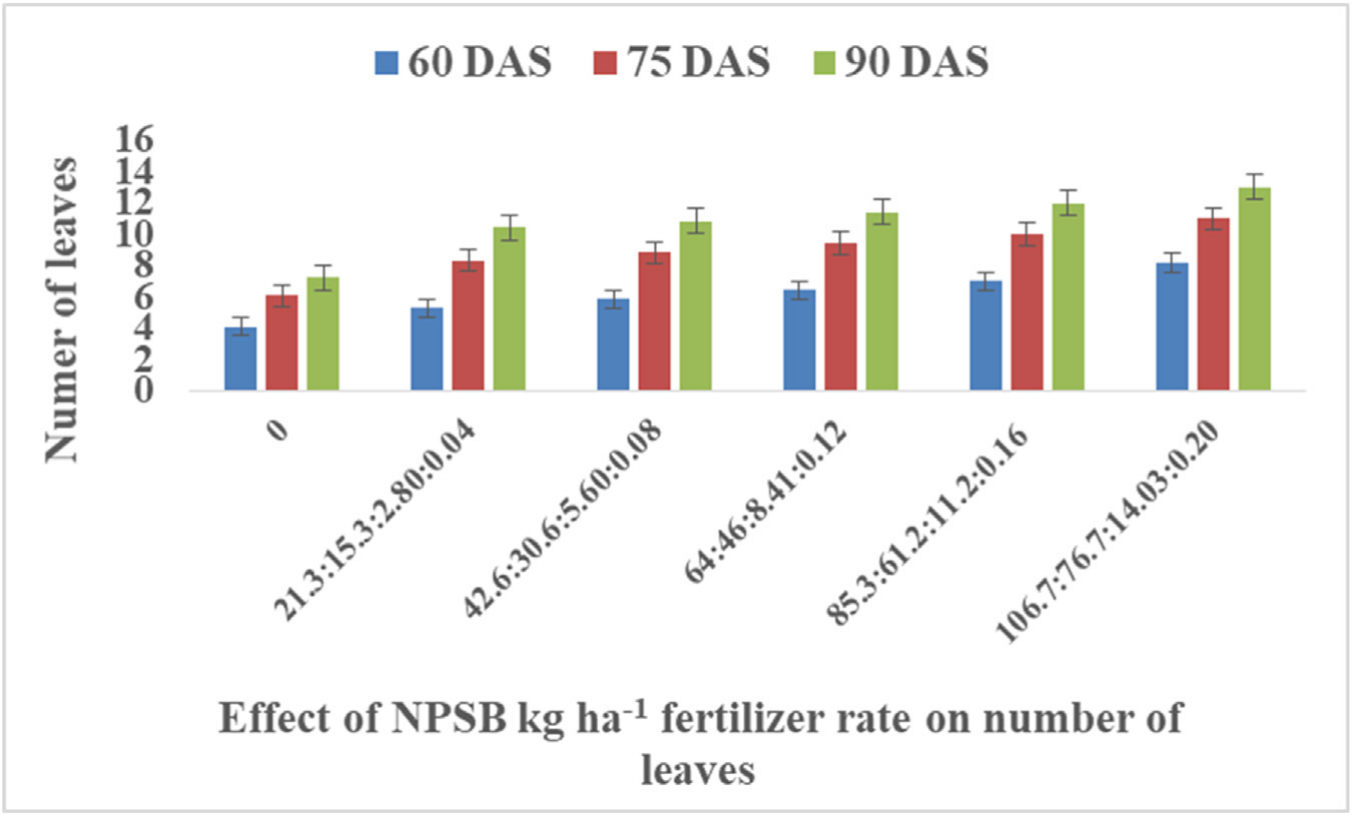
Effects of blended NPSB fertilizer rates on number of leaves between (DAS) days after sowing.

**Fig. 5:**
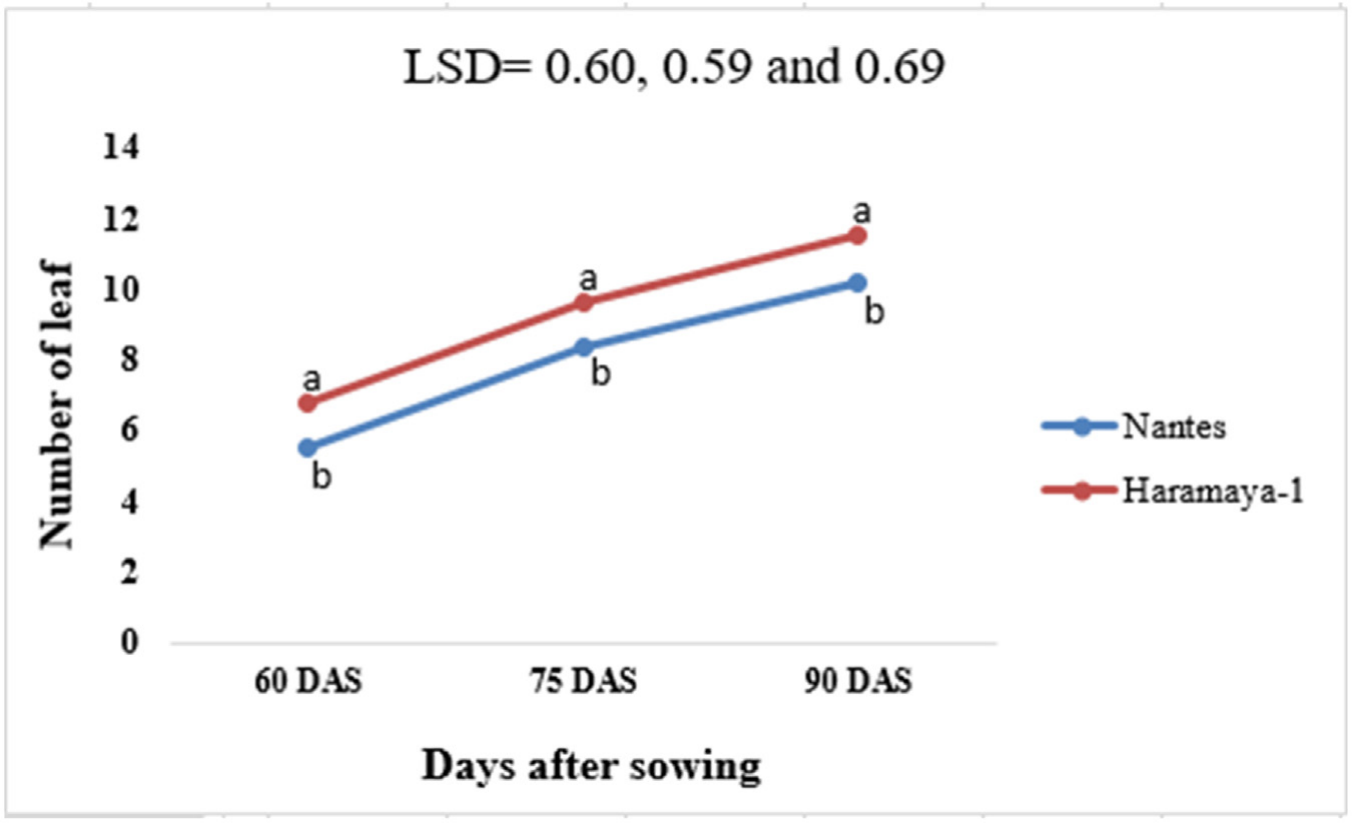
Effects of carrot varieties on the number of leaf.

This dataset was collected in a field experiment conducted under irrigation from December to April 2022 at Teda, Gondar district, Central Gondar Zone, Ethiopia (Fig. 1). Illustrates the main effect means of carrot crop phenology (days to maturity) parameters as affected by NPSB and varieties in Table 1. The main effects of carrot growth (plant height and number of leaves as affected by NPSB and varieties) are shown in Fig. 2, Fig. 3, Fig. 4, Fig. 5, respectively. Interaction effect means of root fresh weight (RFW) and root length (RL) of carrot as affected by NPSB blended fertilizer rates and varieties in Table 2. The main effect of blended NPSB fertilizer rates and varieties on the root diameter (RD) and root volume (RV) of carrots is shown in Table 3. Interaction effect means of root dry matter (RDM) of carrot as affected by blended NPSB fertilizer rates and varieties in Table 4. Main effect means of blended NPSB fertilizer rates and varieties on aboveground biomass (AGBM) of carrots are shown in Table 5. The interaction effect means the marketable yield of carrot as affected by blended NPSB fertilizer rates and varieties in Table 6. The main effect of blended NPSB fertilizer rates and varieties on the unmarketable root yield of carrot is shown in Table 7. The interaction effect means the total yield of carrot as affected by blended NPSB nutrient levels and varieties in Table 8.

**Table 1:** Main effect means of blended NPSB fertilizer rates and varieties on days to maturity.

NPSB (kg ha^−1^)	DM (days)
0:0:0:0	91.55^e^
21.3:15.3:2.80:0.04	97.33^d^
42.6:30.6:5.60:0.08	101.91^c^
64:46:8.4:0.12	105.28^c^
85.3:61.2:11.2:0.16	111.31^b^
106.7:76.7:14.03:0.20	115.61^a^
LSD	3.86***
**Varieties**	
Haramaya-1	102.53^b^
Nantes	105.13^a^
LSD	2.23*

CV (%)	2.23

Means followed by the same letter(s) in a column and row are not significantly different. *, **, ***, significant at P≤0.05, P≤0.01and P≤0.001, respectively.

**Table 2:** Interaction effect means of root fresh weight (RFW) and root length (RL) of carrot as affected by NPSB blended fertilizer rates and varieties.

Treatments	RFW plant^−1^ (g)		RL (cm)	
NPSB rate kg ha^−1^	Haramaya-I	Nantes	Haramaya-I	Nantes
0:0:0:0	71.88^g^	67.80^g^	12.82^f^	12.06^f^
21.3:15.3:2.80:0.04	93.02^f^	92.75^f^	15.03^de^	13.93^ef^
42.6:30.6:5.60:0.08	104.73^e^	102.97^e^	17.46^c^	15.08^de^
64:46:8.4:0.12	144.57^ab^	137.71^c^	18.31^c^	16.62^cd^
85.3:61.2:11.2:0.16	146.49^a^	140.69^bc^	23.36^a^	18.51^bc^
106.7:76.7:14.03:0.20	137.67^c^	123.11d	20.35^b^	17.79^c^

LSD	5.44*		1.91*	

CV (%)	2.83		6.73	

Means followed by the same letter(s) in a column and row are not significantly different. *, **, ***, significant at P≤0.05, P≤0.01and P≤0.001, respectively.

**Table 3:** Main effect means of blended NPSB fertilizer rates and varieties on root diameter (RD) and root volume (RV) of carrot.

NPSB (kg ha^−1^)	RD (cm)	RV (ml)
0:0:0:0	2.13^c^	53.5^e^
21.3:15.3:2.80:0.04	2.26bc	76.5d
42.6:30.6:5.60:0.08	2.56b	81.6c
64:46:8.4:0.12	3.46^a^	97.45^b^
85.3:61.2:11.2:0.16	3.73^a^	114.16^a^
106.7:76.7:14.03:0.20	3.61^a^	107.26^a^
LSD	0.31***	9.62***
**Varieties**		
Haramaya-I	3.1^a^	93.2^a^
Nantes	2.82^b^	85.15^b^
LSD	0.18**	5.55**

CV (%)	8.78	9.01

Means followed the same letter(s) in a column and row are not significantly different. *, **, ***, significant at P≤0.05, P≤0.01and P≤0.001, respectively.

**Table 4:** Interaction effect means of root dry matter (RDM) of carrot as affected by blended NPSB fertilizer rates and varieties.

Treatments	RDM (%)	
NPSB rate kg ha^−1^	Haramaya-I	Nantes
0:0:0:0	6.33^i^	5.91^hi^
21.3:15.3:2.80:0.04	7.86^fg^	7.26^gh^
42.6:30.6:5.60:0.08	9.50^de^	8.23^efg^
64:46:8.4:0.12	10.39^cd^	8.73^ef^
85.3:61.2:11.2:0.16	14.83^a^	10.93^c^
106.7:76.7:14.03:0.20	13.03^b^	11.36^c^

LSD	1.30***	

CV (%)	6.73	

Means followed the same letter(s) in a column and row are not significantly different. *, **, ***, = significant at P≤0.05, P≤0.01and P≤0.001, respectively.

**Table 5:** Main effect means of blended NPSB fertilizer rates and varieties on aboveground biomass (AGBM) of carrot.

NPSB rate kg ha^−1^)	AGBM(t ha^−1^)
0:0:0:0	37.57^e^
21.3:15.3:2.80:0.04	45.71^d^
42.6:30.6:5.60:0.08	60.79^c^
64:46:8.4:0.12	65.47^c^
85.3:61.2:11.2:0.16	75.86^b^
106.7:76.7:14.03:0.20	81.24^a^
LSD	5.25***
**Varieties**	
Haramaya-I	63.10^a^
Nantes	59.12^b^

LSD	3.03*
CV (%)	7.18

Means followed the same letter(s) in a column and row are not significantly different. *, **, ***, significant at P≤0.05, P≤0.01and P≤0.001, respectively.

**Table 6:** Interaction effect mean of marketable root yield of carrot as affected by blended NPSB fertilizer rates and varieties.

Treatments	MRY(t ha ^−1^)	
NPSB rate kg ha^−1^	Haramaya-I	Nantes
0:0:0:0	22.13^f^	21^f^
21.3:15.3:2.80:0.04	28^e^	25.23^ef^
42.6:30.6:5.60:0.08	35.63^c^	28.83^de^
64:46:8.4:0.12	44.03^b^	33.43^c^
85.3:61.2:11.2:0.16	52.26^a^	36.33^c^
106.7:76.7:14.03:0.20	44.8b	32.73^cd^

LSD	4.4***	

CV (%)	7.71	

Means followed the same letter(s) in a column and row are not significantly different. *, **, ***, significant at P≤0.05, P≤0.01and P≤0.001, respectively.

**Table 7:** Main effect means of blended NPSB fertilizer rates and varieties on unmarketable root yield of carrot.

NPSB rate kgha^−1^	UMRY (t ha^−1^)
0:0:0:0	9.13^a^
21.3:15.3:2.80:0.04	7.61^b^
42.6:30.6:5.60:0.08	7.45^b^
64:46:8.4:0.12	5.78^c^
85.3:61.2:11.2:0.16	3.75^e^
106.7:76.7:14.03:0.20	4.61^d^
LSD (0.05)	0.79***
**Varieties**	
Haramaya-1	5.76^b^
Nantes	7.02^a^

LSD	0.46***
CV (%)	10.43

Means followed the same letter(s) in a column and row are not significantly different. *, **, ***, significant at P≤0.05, P≤0.01and P≤0.001, respectively.

**Table 8:** Interaction effect means of total root yield (TRY) of carrot as affected by blended NPSB fertilizer rates and varieties.

Treatments	TRY(t ha^−1^)	
NPSB rate Kgha^−1^	Haramaya-1	Nantes
0:0:0:0	32.23^gh^	30.5^h^
21.3:15.3:2.80:0.04	35.63^efg^	32.83^gfh^
42.6:30.6:5.60:0.08	42.60^c^	36.76^def^
64:46:8.4:0.12	49.03^b^	40.00^cde^
85.3:61.2:11.2:0.16	55.36^a^	40.73^cd^
106.7:76.7:14.03:0.20	48.23b	38.53^cde^

LSD	4.40***	

CV (%)	6.48	

Means followed by the same letter(s) in a column and row are not significantly different. *, **, ***, significant at P≤0.05, P≤0.01and P≤0.001, respectively.

The dataset presented in this article shows that the root yield of carrots was significantly and positively correlated with most of the growth and yield parameters, as shown in Pearson's correlation matrix in Table 9. The partial budget-analyzed dataset for root yield of carrots as influenced by NPSB and varieties is also presented in Table 10.

**Table 9:** Simple correlations (r) among growth, root characteristics and yield of carrot under different rates of blended NPSB fertilizer rate and varieties.

	DM	PH	LN	AGBM	RFW	RL	RD	RV	RDM	MRY	UMRY	TRY
DM	1											
PH	0.65***	1										
LN	0.65***	0.82***	1									
AGBM	0.83***	0.80***	0.80***	1								
RFW	0.81***	0.78***	0.77***	0.87***	1							
RL	0.73***	0.77***	0.79***	0.85***	0.86***	1						
RD	0.81***	0.71***	0.75***	0.85***	0.92***	0.83***	1					
RV	0.86***	0.80***	0.81***	0.87***	0.90***	0.89***	0.87***	1				
RDM	0.78***	0.77***	0.83***	0.88***	0.83***	0.92***	0.83***	0.88***	1			
MRY	0.66***	0.77***	0.78***	0.80***	0.87***	0.92***	0.85***	0.83***	0.88***	1		
UMRY	-0.75***	-0.80***	-0.79***	-0.82***	-0.88***	-0.89***	-0.87***	-0.90***	-0.86***	-0.87***	1	
TRY	0.61***	0.73***	0.74***	0.77***	0.83***	0.90***	0.81***	0.80***	0.85***	0.98***	-0.80***	1

DM = days to maturity, PH = plant height, LN = leaf number, AGBM = above ground biomass, RFW = root fresh weight, RL = root length, RD = root diameter, RV = root volume, RDM = root dry matter, MRY = marketable root yield, UMRY = unmarketable root yield, TRY = total root yield, ns,*, **, ***, significant at P≤0.05, P≤0.01and P≤0.001, respectively.

**Table 10:** Partial budget, carrot variety and blended NPSB fertilizer rates experiment.

Varieties	NPSB rate (kg ha^−1^)	MY (t ha^−1)^	AMY (t ha^−1)^	GB (ETB ha^−1)^	TVC (ETB ha^−1^)	NB (ETB ha^−1^)	MRR (%)
Haramaya-I	0:0:0:0	22.10	19.92	498000	189	497801	-
Nantes	0:0:0:0	21.00	18.9	472500	199.2	472311	D
Nantes	21.3:15.3:2.80:0.04	25.23	22.71	567750	1769.9	565980	4312
Haramaya-I	21.3:15.3:2.80:0.04	28.00	25.2	630000	1794.8	628205	249900
Nantes	42.6:30.6:5.60:0.08	28.83	25.95	648750	3348.9	645401	1106
Haramaya-I	42.6:30.6:5.60:0.08	35.63	32.1	802500	3410.1	79909	251,125
Nantes	64:46:8.41:0.12	33.43	30.09	752250	4936.9	747313	D
Haramaya-I	64:46:8.41:0.12	44.03	39.63	990750	5032.3	985718	55845
Nantes	85.3:61.2:11.2:0.16	36.33	32.7	817500	6494.4	811006	D
Haramaya-I	85.3:61.2:11.2:0.16	52.26	47.03	1175750	6637.7	1169112.3	11423
Nantes	106.7:76.7:14.03:0.20	32.73	29.46	736500	8023.8	728476	D
Haramaya-I	106.7:76.7:14.03:0.20	44.80	40.32	1008000	8132.4	999868	D

MY= marketable yield, AMY = adjusted marketable yield, GB = gross benefit, TVC = total variable cost, = NB net benefit, MRR = marginal rate of return and D = dominated.

**Plant height**

## 4 Experimental Design, Materials and Methods

Two carrot varieties (Nantes and Haramaya-I) and blended fertilizer, NPSB (18.9 N-37.7 P2O5-6.95S-0.1B) and urea (46% N), were used. The varieties were collected from the Holeta agricultural research center for Haramaya-I and the agricultural inputs delivery shop center for Nantes varieties. The experiment consisted of a factorial combination of two carrot varieties (Nantes and Haramaya-I) and six NPSB fertilizer levels (0:0:0:0, 21.3:15.3:2.80:0.04, 42.6:30.6:5.60:0.08, 64:46:8.41:0.12, 85.3:61.2:11.2:0.16, and 106.7:76.7:14.03:0.20 kg ha-1), which were arranged in a randomized complete block design (RCBD) with three replications. The levels of blended fertilizer were set based on the recommended fertilizer for carrots (100 kg DAP and 100 kg urea ha-1) [3]. There were a total of 36 experimental plots. The size of each plot was 1.8 m2 (1.2 m × 1.5 m), with 6 rows per plot and 24 plants per row. The distance between block and plot was 1.5 m and 1 m, respectively.

The outer single row on both sides of the plot and one plant at both ends of the rows were considered border plants. Hence, the net plot area was 1.1 m^2^ (1 m × 1.1 m).

All rates of NPSB were applied during sowing. Since nitrogen is a mobile element, urea was applied in two split doses: 50% during planting and the remaining 50% thirty-five days after emergence. Thinning was done 30 and 40 days after emergence to maintain the spacing of 5 cm between plants. Irrigation water was supplied from the beginning to the end by the surface irrigation method at 2-day intervals until the seedling established well, and then at 7-day intervals during the experimental period. Plots were irrigated to refill field capacity. Earthing-up was done every two weeks to cover and protect roots from sunlight. All other management practices, including weeding and hoeing, were done as per recommendation.

## 5 Data Collected

### 5.1 Phenology parameter

The number of days from sowing to maturity was counted and recorded. Plants showed yellowing of leaves at the time of reaching physiological maturity, and harvesting of carrot roots was done [5].

### 5.2 Growth parameters

Plant heights (cm) were measured. Ten randomly selected and tagged plants were measured from ground level to the top of the shoot at 60, 75, and 90 days after sowing (DAS) using a ruler. The number of leaves was counted from ten randomly selected and tagged plants from each experimental plot at 60, 75, and 90 days after sowing (DAS).

### 5.3 Root characteristics

Root fresh weight (g) was determined by measuring the fresh weight of 10 randomly collected carrot roots directly after harvesting. The length of the roots of ten randomly selected and tagged plants was measured from the base to the marked apex by a ruler, and the mean length of the roots was calculated at harvesting. The diameter of the root of ten sample plants was measured approximately 2.0 cm below the shoulder by a Vernier caliper, and the average diameter of the root was recorded. The average root volume was taken from 10 randomly selected plants for each treatment by immersing the roots in a beaker containing a known amount of water. The volume of the root was determined by observing the displacement of the water by the root, so that the difference was taken as the volume of the root. Root dry matter (%) was determined by oven-drying carrot root. 100 g of fresh root weight was taken from each plot and oven-dried at a temperature of 80 °C for 72 h or to a constant weight, and the weight was measured using a balance. Dry matter = $\frac{dry\;weight\;of\;sample\;}{fresh\;weight\;of\;sample}$ × 100

### 5.4 Yield parameters

The aboveground biomass was measured after the harvesting of carrots; the biomass was separated from the root (root crown), and its weight was measured using a sensitive balance. Marketable yield (t ha-1) was recorded as roots with no deformities like cracks, forking, disease, or malformation, and those without spots and weighing above 40 grams were selected from each net plot area, and the weight was recorded as marketable yield and converted to t ha-1. Roots that showed root cracks, forking, disease, and malformation and weighed below 40 g were selected from each net plot area, and their weight was recorded as unmarketable yield and converted to t ha-1. The total yield of roots (t ha-1) was measured after harvesting, topping, and cleaning of the roots; the weight of the roots was measured for each net plot. The total yield of the net plot area was converted into t ha-1.

### 5.5 Statistical data analysis

Data were subjected to the Analysis of Variance (ANOVA) using SAS version 9.4 [6] software and general linear model (GML) procedures. Treatment means were separated by the least significant difference (LSD) at 1 or 5% level of significance, depending on the ANOVA result. Correlation analysis was carried out by calculating simple linear correlation coefficients between growth and yield.

### 5.6 Partial budget analysis

An economic analysis was conducted to assess the feasibility of the treatments. Partial budget, dominance, and marginal analysis were used. A partial budget was used to organize experimental data and information about the costs and benefits of various alternative treatments. The partial budget was calculated by using an adjusted marketable yield.

The total costs that vary with treatment are the sum of the costs of yield transport, NPSB, and urea. The costs of NPSB, urea, and application were Birr 18 kg-1, 16 kg-1, and Birr 0.5 kg-1, respectively. Transportation costs were considered to be Birr 0.1 kg-1.

The marketable yield was adjusted downward by 10%, assuming that farmers could get 10% less yield [7]. The 2021-year selling price (Birr 25.00 kg-1) for carrot yield was used in the analysis. Any treatment that has net benefits less than or equal to those of treatment with lower costs was considered dominant and excluded in the marginal analysis. For the majority of situations, the minimum rate of return acceptable to farmers is between 50 and 100% [7]. Therefore, the minimum acceptable marginal rate of return was considered to be 100% in this study.

## Limitations

None.

## Ethical Statement

The current work does not involve human subjects, animal experiments, or any data collected from social media platforms.

## CRediT authorship contribution statement

**Abebaw Mulugeta Andualem:** Conceptualization, Methodology, Software, Writing – original draft, Investigation, Formal analysis. **Yenus Ousman Kemal:** Supervision, Writing – review & editing. **Derajew Asres Mekonen:** Supervision, Writing – review & editing. **Fentahun Asrat Yenet:** Supervision, Writing – review & editing. **Hulushum Woreta Kassa:** Supervision, Writing – review & editing.

## Data Availability

Mendeley DataEffect of NPSB fertilizer rates on growth and yield of carrot (Daucus carrota L.) varieties in Gondar district, Northwestern Ethiopia (Original data).

## References

[1] G. De Lannoy, (2001).Carrot Crop Production in Tropical Africa. R.H. Raemaekers (ed.) Directorate-General for International Corporation, Brussels, Belgium. 480–485.

[2] FAO (Food and Agriculture Organization of the United Nations). 2019. FAOSTAT databases. (URL: http://www.fao.org/faostat/en/#data/QC). Accessed on April 6, 2021.

[3] Mohammed W., Bezu T., Dechassa N., Woldetsadik K., Hailu M., Abebie B. (2014). Registration of “Haramaya I” Carrot (*Daucus carota* L.) variety. East Afr. J. Sci..

[4] J.M. Hart and M.D. Butler, (2003). Seed carrot aboveground biomass and nutrient accumulation.

[5] Hailu S., Seyoum T., Dechassa N. (2008). Effect of combined application of organic P and inorganic N fertilizers on post-harvest quality of carrot. Afr. J. Biotechnol..

[6] SAS 9.4 Output Delivery System: user's Guide, 2024.

[7] CIMMYT. (1988). from agronomic data to farmer's recommendations: an economics training manual. Completely revised edition, CIMMYT, Mexico. D.F. 79.

